# Microstructural Basis of Complex Mechanical Programming in Liquid Crystal Elastomers

**DOI:** 10.1007/s10659-025-10138-4

**Published:** 2025-05-29

**Authors:** Morgan Barnes, John S. Biggins

**Affiliations:** 1 Department of Engineering, University of Cambridge, Trumpington St., Cambridge, CB2 1PZ UK; 2 Present Address: Manufacturing Futures Laboratory, Department of Mechanical Engineering, University College London, London, WC1E 7JE UK

**Keywords:** Nematic elastomer, Complex shape change, Microstructure, Soft-mode, Polydomain, 74A60, 74B99, 74E25, 74F99

## Abstract

Liquid crystal elastomers (LCEs) are actuating soft solids that exhibit large and reversible contractions along the liquid crystal director on heating through the nematic-isotropic transition. Recently, mechanical programming was used to fabricate LCEs that can actuate into arbitrarily complex shapes such as a face. Here, we combine theoretical and experimental observations to explain how such complex mechanical programming works. Crucially, we identify the intermediate pre-programmed state as an isotropic genesis polydomain, which, during programming, can accommodate the imposed strains via a pure soft-mode response enabled by director rotation and laminar microstructures. Second cross-linking fixes these microstructures as preferred but, since they were achieved softly, they do not involve any reconfiguration of the isotropic state, allowing it to be restored on heating. Experimental observations of programmed LCEs reveal both single and double laminate structures, as anticipated by the theory, and also a quantitative match between the 3D deformations that can and cannot be programmed. The set of programmable deformations includes all modest isochoric deformations, explaining why arbitrary shape programming works. Finally, we demonstrate theoretically and experimentally that programming also allows one to engineer samples with desired extents of softness in different directions.

**Supplementary Information:**

The online version contains supplementary material available at 10.1007/s10659-025-10138-4.

## Introduction

Soft solids, such as gels, elastomers, and foams, are unified by their ability to sustain large and reversible deformations in response to slight loads. Correspondingly, soft solids can also deform dramatically in response to non-mechanical stimuli, enabling the burgeoning subject of soft-actuators. Prominent examples include dielectric elastomers [1], swelling gels [2], phase-changing liquid crystal elastomers (LCEs, [3, 4]) and pneumatic baromorphs [5]. In each case, a homogeneous sample of material will deliver a large but uniform spontaneous deformation, such as the isotropic dilation in a swelling gel or uniaxial contraction in a heated LCE. However, just as biology uses differential growth to sculpt complex shapes during morphogenesis [6], one may achieve complex shape changes with soft actuators via a spatial pattern of swelling [7, 8] contraction [9], inflation [5] or dielectric actuation [10]. In such cases, a pattern of actuation must be first designed (often by solving a difficult inverse problem [11–13]) and then a complex fabrication strategy is required to programme the pattern into the material. Furthermore, experimental results for complex programmed shapes like faces are often somewhat disappointing, largely because the fabrication systems can only implement a restricted palate of patterns that do not support a complete solution to the inverse problem. However, a radically different approach was recently demonstrated in nematic LCEs, wherein mechanical programming was used to implement convincing complex shapes without any need for pattern design or complex fabrication [14]. Here we seek to explain why such programming works.

A nematic LCE is a cross-linked rubbery network of LC rods, and actuates via a temperature driven isotropic-nematic phase transition which generates a substantial and reversible extension ($\lambda _{s}\sim 2$) along the alignment direction on cooling to nematic. A key obstacle to creating actuating LCEs is that, if one simply cross-links an isotropic fluid then cools to the nematic, one obtains a polydomain with no global actuation [15]. To create an actuating monodomain, one must thus imprint a preferred alignment into the LCE. From the outset, two broad methods have been used to achieve such imprinting: crosslinking an already aligned nematic fluid [16], and two-step crosslinking in which an initial round of cross-links form a soft network which is then mechanically stretched to drive alignment and then crosslinked a second time [3]. Historically, complex shape-programming has been achieved in the first manner, by imposing a spatial director profile on a nematic fluid using surface anchoring techniques, and then crosslinking to make a programmed LCE [9, 11, 17, 18]. 3D printing of LCEs proceeds very similarly, with viscous forces during extrusion aligning a nematic polymer fluid, which is then cross-linked [19–21]. In this case, creating samples with complex director profiles requires first designing the director profile, then creating a print path that everywhere extrudes along the desired direction [22–24]. Photo-patterning and 3D printing are thus good examples of processes that requires both pattern design and complex fabrication to achieve complex morphing.

The new approach of *Barnes and Verduzco* [14] instead proceeds in the second manner, with an initial round of cross-links forming a soft isotropic network, which is then cooled to nematic, distorted mechanically into a complex shape, and cross-linked a second time. Remarkably, this approach produces an LCE with a complex director profile that reliably actuates between the programmed shape and the original isotropic shape.

At first sight, the success of mechanical programming for complex shapes is quite mysterious. The original mechanical mono-domain protocols, pioneered by Finkelmann, used a constant uniaxial stress field for imprinting [3], but with no expectation (or observation) that the resultant strain is precisely reversed during actuation; indeed programming strains were often far higher than the actuation strains, to ensure the best possible global alignment. Furthermore, by analogy with the work on complex actuation via director design, it appears that the new protocol must create a spatial director profile that exactly reverses the complex programming strain on actuation, but why should it do so? If this is correct, understanding the director pattern chosen by mechanical programming may also enable better explicit director design.

The mechanics of nematic elastomers is greatly enriched by a second consequence of the symmetry-breaking isotropic-nematic phase transition: soft modes of deformation. In brief, on cooling a perfectly isotropic LCE a direction must be chosen for alignment, so there are many degenerate ground states with different material axes for alignment and elongation. Consequently, the (large) deformations that map between these states rotate the director within the LCE but, Goldstone-like, do not cost any energy to impose [25, 26]. Soft modes in turn make nematic LCEs subject to non-soft deformations susceptible to the formation of rapidly oscillating “stripe-domain” microstructures [27–29] which allow the non-soft deformation to be accommodated macroscopically while all local deformations are soft.

In this paper, we explain theoretically and demonstrate experimentally why mechanical programming of complex shapes works, and how it depends critically on soft elasticity and microstrucure. A key clue to the success of complex mechanical programming comes from a small deviation from the original mechanical mono-domain protocols, which undertook the second cross-linking under stress, but in the high temperature isotropic state, [3, 30], leading to a softer more “ideal” LCE [31]. In contrast, during complex programming, the first network is cooled to a nematic polydomain state before being strained and second cross-linked. This difference endows the programming step with the full richness of soft-modes, director rotation and microstructural instabilities. Here we demonstrate that this added richness is what permits complex shape programming: in short, mechanical programming works when the strains imposed can be accommodated by laminar microstructures of soft modes of the original network. This new insight allows us to predict and understand what strains can be programmed, give examples of microstructures that can programme them, and also predict the soft mechanical response of the resultant programmed samples.

## Motivating Experimental Observation

We start with an explicit demonstration of complex mechanical programming. Following [14], samples were prepared from a mixture of di-functional acrylate rods (RM257), a di-functional thiol chain extender (EDDET) and a tetra-functional thiol cross-linker (PETMP). Initially the reagents are prepared in a chloroform solution, and a first round of base-catalyzed thiol-acrylate chemistry occurs, leading to chain extension and cross-linking. This initial network forms in isotropic solution, but upon deswelling and cooling, the network becomes a turbid nematic polydomain. This sample is then mechanically deformed into the programmed shape, and then a second round of (acrylate-acrylate) cross-links are created via UV curing. Actuation is then observed by placing the sample on a hot-plate, to heat it back to isotropic.

**Fig. 1:**
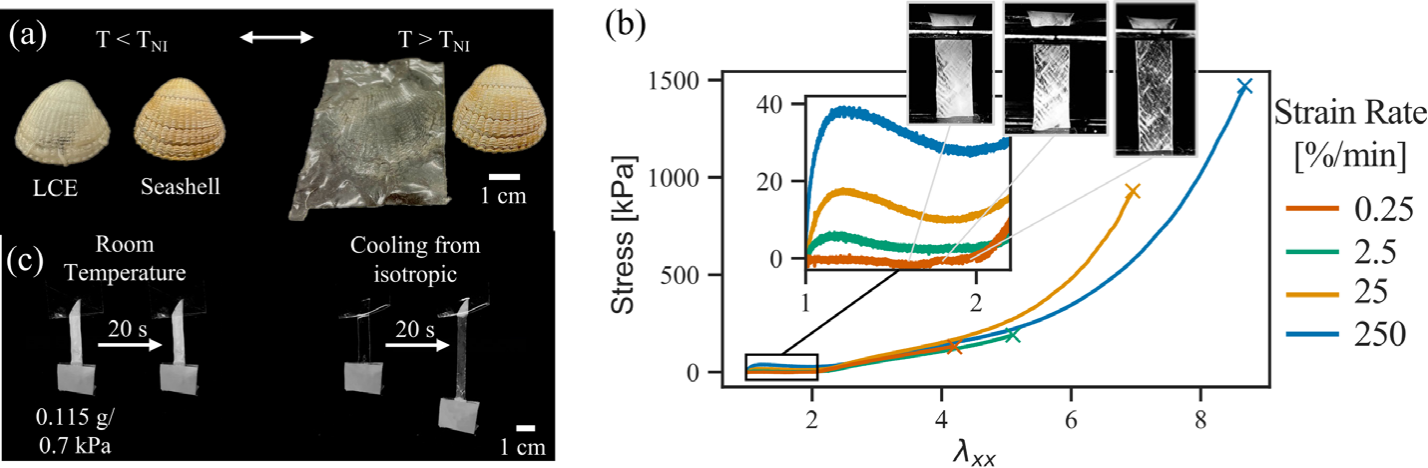
(a) Images of an LCE film that was pressed onto the surface of a seashell prior to UV curing the second network. The resulting film reversibly transitions between a seashell and a flat film upon heating/cooling. (b) Stress-stretch curve of an LCE stretched prior to UV curing. Inset photographs show the microstructure that forms in quasi-equilibrium stretch regimes (0.25%strain/min). (c) Photographs of a polydomain LCE prior to UV curing. With small weights the room temperature LCE does not stretch at short/mid time-scales, but the same LCE stretches/aligns from the weight when cooled from the isotropic

In Fig. 1(a) we demonstrate an example of complex shape programming, which combines bend and stretch to achieve morphing of a thin sheet into the complex surface of a seashell.

To confirm our general hypothesis that programmability is related to soft elasticity, we obtained stress-strain curves for the uniaxial extension of the turbid nematic poly-domain samples (after first crosslinking, but before second crosslinking) at increasing strain rates. In Fig. 1(b), we see that at very slow strain-rates the polydomain samples elongate very softly, at essentially zero stress, until they reach an elongation of $\lambda \sim 2$, corresponding to the clear mono-domain state. Higher elongations then produce an increasing stress response, as would be typical of a conventional elastomer. Correspondingly, *Barnes and Verduzco* observed in their original work that with this LCE system, one can successfully program all uniaxial stretches with $\lambda \le 2$ (the soft elastic region), and the link between the soft elastic limit and the limit of uniaxial programmability was also made in a very recent independent study [32]. Conversely, in both previously studies, although over-stretching during programming was observed to make good globally aligned monodomain samples, they relaxed in shape somewhat after second cross-linking and do not recover the original isotropic shape on heating, so, although they can be good actuators, they are not faithful to the programming.

Interestingly, during our slowest stretching experiments, microstructure is visible in the LCE even in simple reflected light photographs, with cross-hatch patterns, turbid polydomain regions and aligned clear mono-domain regions all evident. Overall this data confirms the presence of soft elasticity in the initial system, and the relationship between the extent of soft elasticity, the formation of microstructure and the extent of programmability.

Additionally, we observe that when stretching is imposed at higher-strain rates, the stress plateau for “soft” behavior and director reorientation is elevated, meaning higher stresses are needed to achieve the same strains. Practically this means an LCE will not strain when subjected to small stresses for short-times, as shown in Fig. 1c where a small weight causes no appreciable stretching over 4 h, but is sufficient to stretch the sample into a monodomain if left overnight. However, as also seen in Fig. 1c, one can achieve the same deformation and reorientation at this same force scale in just a few seconds by heating the LCE to isotropic and then allowing it to cool back to nematic under load. This acceleration reflects the well-reported softening of thiol-acrylate LCEs as they approach the nematic/isotropic phase transition ($T_{ni}$) from below, [33], which is followed by rapid stiffening on further heating in the isotropic state. During cooling from the isotropic under stress, rapid deformation and reorientation thus occurs just below the transition temperature. Correspondingly, during programming of complex shapes, it is often faster and more reliable to deform the LCE while isotropic, then cool to nematic in the complex shape, a strategy that we adopted for programming the seashell in Fig. 1(a).

## Mechanical Microstructure Selection

To explain the success of complex mechanical programming, we focus on the isotropic-nematic transition that occurs in first network. Initial cross-linking happens in isotropic solution, leading to the formation of an isotropic network. After deswelling, we thus have a simple isotropic rubber, which forms the reference state for our subsequent analysis. We take this rubber as an incompressible neo-Hookean, meaning that if it is subject to a deformation moving the material point $\mathbf{R}$ to $\mathbf{r}(\mathbf{R})$, and resultant deformation gradient $\Lambda =\nabla \mathbf{r}$, then it stores elastic energy $W_{NH}(\Lambda )={\textstyle \frac{1}{2}}\mu (\operatorname{Tr}{(\Lambda \cdot \Lambda ^{T})}-3)$, where $\mu $ is shear modulus, and the constraint of incompressibility requires $\mathrm{Det}{\Lambda}=1$.

**Fig. 2:**
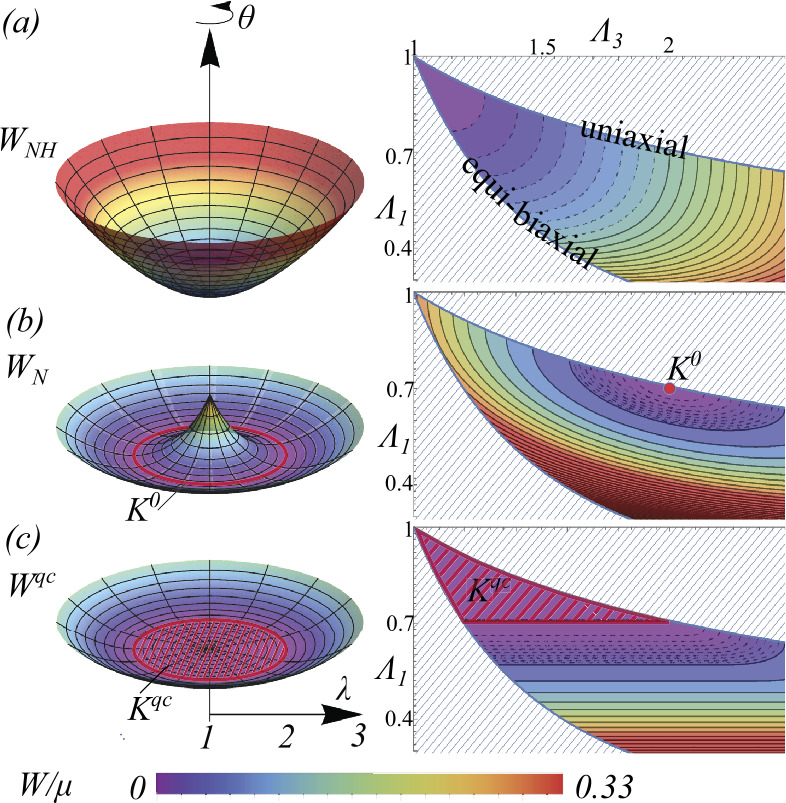
Elastic energy functions for (a) Neo-Hookean rubber $W_{NH}$ (b) an ideal nematic elastomer subject to homogeneous deformation $W_{N}$, (c) an ideal nematic elastomer after microstructural relaxation $W^{qc}$. On the left are surface plots of the respective energy functions for a uniaxial deformation of magnitude $\lambda $, with the axis of deformation in the $x-y$ plane at an angle $\theta $ to the $x$ axis (i.e. in an $x-y-z$ basis, $\Lambda =R_{z}(\theta ) \cdot \mathrm{diag}(\lambda , 1/\sqrt{ \lambda},1/\sqrt{\lambda}) \cdot R_{z}(\theta )^{T}$ where $R_{z}(\theta )$ is a rotation by $\theta $ around $z$). On the right is a contour plot of the energy as a function of the smallest ($\Lambda _{1}$) and largest ($\Lambda _{3}$) principal stretches. The nematic energies are plotted with $\lambda _{s}=2$. $K^{0}$ indicates the low energy monodomain states, while $K^{qc}$ indicates states that become low energy after formation of microstructure. Vertical scale and colour scale of $W_{NH}$ are compressed by factor of 7.5 to facilitate comparison with $W_{N}$ and $W^{qc}$, which are both much smaller in the plotted domain

An intuitive 2D surface plot of the neo-Hookean energy is provided in Fig. 2(a)i, which shows the energy of incompressible uniaxial elongations by a factor of $\lambda \ge 1$, applied along a material direction in the $x-y$ plane making an angle $\theta $ with $x$. Intuitively, all directions of elongation are equivalent, so the energy forms a surface of revolution, with a single global minimum at $\lambda =1$, the undeformed state. More formally, the elastic energy of any isotropic material can be written as a function of the three principal stretches $\Lambda _{i}$ (i.e. the eigenvalues of $\sqrt{\Lambda ^{T}\cdot \Lambda}$ ordered such that $\Lambda _{1} \le \Lambda _{2} \le \Lambda _{3}$). Given rubber is also incompressible ($\Lambda _{2}=1/(\Lambda _{1} \Lambda _{3})$) this means any rubber energy can in fact be written as a function of just two stretches: $\Lambda _{1}<1$ and $\Lambda _{3}>1$, and a plot of a the energy function in $\Lambda _{1}$, $\Lambda _{3}$ space will thus completely describes the function. For the neo-Hookean energy, this change of variables gives $W_{NH}(\Lambda _{1}, \Lambda _{3})={\textstyle \frac{1}{2}}\mu ( \Lambda _{1}^{2}+1/(\Lambda _{1} \Lambda _{3})^{2}+\Lambda _{3}^{2}-3)$, and the corresponding plot is provided in Fig. 2(a)ii. The global minimum is now seen at the undeformed point in the top left, $\Lambda _{1}=\Lambda _{3}=1$. We also note that such functions are also always only defined in the sub-region of the $\Lambda _{1}-\Lambda _{3}$ plane sandwiched between the uniaxial elongation line ($\Lambda _{1}=\Lambda _{2}=1/\sqrt{\Lambda _{3}}$) and the equibiaxial deformation line ($\Lambda _{3}=\Lambda _{2}=1/\sqrt{\Lambda _{1}}$, aka uniaxial compression), as outside this region the implied $\Lambda _{2}$ is not between $\Lambda _{1}$ and $\Lambda _{3}$.

Upon cooling through $T_{ni}$ nematic order forms along a local (unit) director ${\mathbf{N}}$ (capitalized to denote a reference state vector) leading to a local spontaneous uniaxial elongation by $\lambda _{s}$ along ${\mathbf{N}}$. This spontaneous deformation may be written as the spontaneous deformation gradient $\Lambda _{s}({\mathbf{N}})=(\lambda _{s}-1/\sqrt{\lambda _{s}}) { \mathbf{N}}{\mathbf{N}}+ 1/\sqrt{\lambda _{s}} I$, $I$ being the 3x3 identity matrix. To construct the nematic elastomer energy function, we first consider that a nematic LCE with fixed ${\mathbf{N}}$ would simply behave as a standard neo-Hookean rubber, but with this new elongated state as the relaxed state, leading us to ascribe the energy $W_{NH}(\Lambda .\Lambda _{s}^{-1}({\mathbf{N}}))$ to a general deformation $\Lambda $ from the original isotropic state. As expected, this energy is now minimized by $\Lambda =\Lambda _{s}({\mathbf{N}})$. Secondly we consider that the director ${\mathbf{N}}$ is not fixed in the LCE, but may rotate to minimize the elastic energy, giving a final energy that is a function only of deformation gradient:

1 $$ W_{N}(\Lambda ) = \min _{{\mathbf{N}}}W_{NH}(\Lambda \cdot \Lambda _{s}^{-1}({ \mathbf{N}})) \mathrm{, \ \ \ \ } \mathrm{Det}({\Lambda})=1. $$

This energy is sometimes known as the “ideal” energy for nematic elastomers, and may also be derived directly from statistical mechanics [4, 34], giving the additional insight that $\lambda _{s}=r^{1/3}$, where $r$ is the anisotropy ratio of the polymer random walks induced by nematic order. We note that the celebrated “trace formula” produced by the microscopic approach is traditionally written as $\min _{{\mathbf{n}}} W_{NH}(\Lambda _{s}^{-1}({\mathbf{n}}) \cdot \Lambda )$, where ${\mathbf{n}}$ is the physical director written as a unit vector in the final-state, whereas our phenomenological justification leads to the above form containing this director’s reference state counterpart ${\mathbf{N}}$. However, by writing both neo-Hookean functions out, one may observe that they are equivalent provided ${\mathbf{N}}$ is derived from the physically observable ${\mathbf{n}}$ via the same rotation ${\mathbf{N}}=R {\mathbf{n}}$ as relates the left and right Cauchy-Green tensors $\Lambda \Lambda ^{T}= R^{T} \Lambda ^{T}\Lambda R$. Furthermore, minimization of either form of the energy over the director requires it to lie along the eigenvector of the (left-or right respectively) Cauchy-Green strain with the largest principal stretch, $\Lambda _{3}=\max \{\Lambda _{1},\Lambda _{2},\Lambda _{3}\}$, leading to the director-free form [29]

2 $$ W_{N}(\Lambda ) = {\textstyle \frac{1}{2}}\mu \left ( \Lambda _{1}^{2} \lambda _{s} + \Lambda _{2}^{2} \lambda _{s}+ \Lambda _{3}^{2}/ \lambda _{s}^{2} -3\right ), \mathrm{\ \ \ \ } \Lambda _{1} \Lambda _{2} \Lambda _{3}=1. $$

Predictably, after minimizing over director, the energy function is again in the form for an isotropic solid, reflecting the symmetry of the original hot parent state. Further minimizing the energy subject to the constraint of incompressibility, $\Lambda _{1}\Lambda _{2}\Lambda _{3}=1$, gives the minimum $\Lambda _{3}=\lambda _{s}$, $\Lambda _{1}=\Lambda _{2}=1/\sqrt{\lambda _{s}}$, i.e. the expected spontaneous deformation but along any axis, explicitly revealing the degenerate set of ground states that generate soft modes. Formally, we may write the set of zero energy deformations as [29]

3 $$ K^{0} =\left \{ \Lambda \in \mathbb{M}^{3 \times 3} \mathrm{\ s.t.\ } \Lambda _{1} = \lambda _{s}^{-1/2},\Lambda _{3} = \lambda _{s}, \mathrm{Det}(\Lambda )=1\right \} $$

In Fig. 2(b) we provide an analogous pair of energy plots for $W_{N}(\Lambda )$, to show how it differs to the standard neo-Hookean energy. The intuitive surface plot now takes on a “Mexican-hat” character, with a degenerate ring of minima corresponding to elongations by $\lambda _{s}$ but in any direction. On the more complete $\Lambda _{1}-\Lambda _{3}$ plot, the minimum is still seen a single point, but now a finite distance along the uniaxial line, again conveying that all such uniaxial deformations are global minima.

The energy $W_{N}(\Lambda )$ was initially developed to describe mono-domain nematic LCEs. However, the development above clarifies that is also the appropriate energy function for isotropic-genesis polydomain LCEs [35], as used in mechanical programming. However, this leads to an apparent contradiction: the polydomain does not deform macroscopially on cooling into the nematic state, but simply becomes turbid, yet the undeformed state $\Lambda =I$ is no longer the minimum of the energy. Why doesn’t the LCE spontaneously elongate into a global minima in the brim of the Mexican hat? The resolution is that the LCE does in fact do this, but in a microstructural domain structure, with different directions for elongation/alignment in different domains, such that, although each domain actuates, there is no global deformation. This realization suggests that we should construct a new microstructurally relaxed energy for the polydomain LCE, in which the deformation $\Lambda $ from the original isotropic reference state is only imposed on average (or macroscopically), while allowing complex microstructural deformations within. Formally, we thus say that, if we impose a macroscopic deformation $\Lambda $ from the original isotropic reference state, the network will distort into final positions $\mathbf{r}(\mathbf{R})$ that minimize the above ideal energy $W_{N}$, subject to meeting the imposed deformation macroscopically:

4 $$\begin{aligned} W^{qc}(\Lambda )=\!\!\min _{ \substack{\mathbf{r}(\mathbf{R}) \, \mathrm{{s.t.} }\\ \left < \nabla \mathbf{r} \right > =\Lambda} }\frac{1}{V_{\Omega}}\int _{\Omega }W_{N}( \nabla \mathbf{r}) \mathrm{d} V. \end{aligned}$$

For simple homogeneous hyper-elastic energies such as the neo-Hookean this minimization (known as quasi-convexification, hence $W^{qc}$) simply gives $\nabla \mathbf{r}=\Lambda $, meaning the material deforms locally exactly following the macroscopic deformation. However, for the nematic energy, $W_{N}$, the adoption of microstructural deformations often saves energy. In particular, a much larger set of macroscopic deformations than $K^{0}$ can be achieved at zero energy, $W^{qc}(\Lambda )=0$, by the formation of microstructures that are everywhere locally within $K^{0}$. Remarkably, this microstructural relaxation problem has been solved analytically in previous studies focused on nematic monodomains [29]. The full set of deformations that can be made zero energy in this way, $W^{qc}(\Lambda )=0$ is simply [29]

5 $$\begin{aligned} K^{qc} =\left \{ \Lambda \in \mathbb{M}^{3 \times 3} \mathrm{\ s.t.\ } \Lambda _{1} \ge \lambda _{s}^{-1/2},\Lambda _{3} \le \lambda _{s}, \mathrm{det}(\Lambda )=1\right \} \end{aligned}$$

which, informally, means that all incompressible deformations less severe that $\Lambda _{s}$ may be made zero energy by the adoption microstructural deformations. The non-zero energies for other deformations are also known (some involving microstructure, and some not), allowing us to again provide an analogous pair of energy plots for $W^{qc}(\Lambda )$, as shown in Fig. 2(c). In particular, we observe on both plots that the set of zero energy states ($K^{qc}$) now includes a whole region of deformation space extending from the “brim” of the Mexican hat ($K^{0}$) all the way to the unreformed state ($\Lambda =I$). Thus the observed polydomain state that forms on cooling, which is macroscopically undeformed, is in fact at a global minimum of the relaxed energy.

Furthermore, all states in $K^{qc}$ are energetically equivalent, meaning that the sample is “supersoft” and may be distorted between any two such states with the deformation accommodated at zero energy via director rotation and microstructural evolution [35–37]. Indeed, in our initial experimental observations, we observed such a sample can be uniaxially stretched by a factor of two at almost zero stress, at which point it becomes transparent (indicating global alignment), and additional stretches are resisted by significant elastic stress (Fig. 1(b)). Furthermore, in practice, deformations within the zero stress region often do not recover at all upon release, confirming that states within $K^{qc}$ are essentially degenerate. However, if a sample distorted to any point within $K^{qc}$ is then heated back to isotropic, the energy returns to the original neo-Hookean energy minimized (locally and globally) by $\Lambda =I$, and correspondingly the sample returns, locally and globally, to its original shape. This one-way “shape-memory” effect is directly analogous to that generated by austenite-martensite transitions in shape-memory alloys [38].

## Second Cross-Linking

To move from shape-memory to programmed actuation, the isotropic genesis polydomain is deformed into its target shape, and then cross-linked a second time. To understand the effect of this second cross-linking, it is helpful to recall the simplest microscopic theory of LCEs, which assumes long (Gaussian) chains of freely jointed rods. This limit gives a total free energy that is the sum of a rubber-like term from the network strands and a standard Landau-de-Gennes (liquid-nematic) type term from the rods [4]:

6 $$ W(Q, {\mathbf{n}}, \Lambda )=\frac{1}{2} n_{s} k_{B} T \operatorname{Tr}{(\Lambda \cdot \Lambda ^{T}\cdot \Lambda _{s}^{-2}({\mathbf{n}}))} + n_{R}k_{B} T f_{LdG}(Q,T), $$

where $Q$ is the standard scalar order parameter for liquid crystals, $n_{s}$ number-density of cross-links, $n_{R}$ the number-density of rods, while, for a freely jointed chain, the preferred spontaneous elongation relates to the degree of order as $\lambda _{s}=(1+2Q)/(1-3Q)$ [4] In principle, the elastic constitutive law of a nematic LCE, $W_{N}(\Lambda )$, is given by minimizing the above over $Q$ and ${\mathbf{n}}$ at fixed imposed deformation $\Lambda $. However, in a soft LCE, we expect many rods per chain, $n_{s} \ll n_{R}$, so that the Landau-de-Gennes rod energy has a higher scale than the rubber one. In this approximation, minimization over $Q$ is dominated by $f_{LdG}(Q,T)$, so that $Q(T)$ follows the same form as the uncross-linked nematic fluid, which in turn fixes $\lambda _{s}(Q)$ to a simple function of temperature. These first order theoretical results suggest that the cross-linking has minimal effect on the extent of the nematic order, or on the polymer chain anisotropy induced by the order, which both remain the same as in the polymer melt. After minimizing on $Q$ to obtain $\Lambda _{s}$, minimization over ${\mathbf{n}}$ brings us exactly to Eq. (2), with the identification $\mu =n_{s} k_{B} T$. Of course, the light-cross-linking limit is an approximation, and, for example, in reality $T_{ni}$ does shift a little upon cross-linking [39], and such effects can be predicted within this framework by minimizing both elastic and LdG terms over $Q$ simultaneously [4], however such effects are typically small in elastomers, and, to a first approximation, can be safely ignored.

Within these microscopic approximations, the sole influence of a second round of cross-linking is to increase the shear modulus. Interestingly, provided the LCE is still soft and rubbery, we do not predict any substantial change in $Q(T)$ which will still be liquid-like, nor of $\lambda _{s}(Q)$. Because the spontaneous deformation is unchanged, we thus also predict the material will simply continue to behave in the same shape-memory manner as before, with the same set of deformations in $K^{qc}$, made zero energy by the same microstructures.

This conclusion accords with early theory by de-Gennes [40] and Warner [4, 41, 42] showing that such perfect Gaussian LCEs generally show the same behavior whether crosslinked in the isotropic or nematic state. In particular, within Gaussian statistics, even if an LCE is crosslinked in the nematic state, all memory of the director at cross-linking is lost upon heating to the isotropic. Thus, even LCEs cross-linked in the nematic state are described by $W_{N}(\Lambda )$, and are expected to show perfectly soft elastic deformations associated with director rotations [26]: indeed the microstructural relaxation of $W_{N}$ was originally motivated by LCE monodomains. In this instance, the simplest LCE theory has a qualitative problem. An LCE crosslinked in a nematic monodomain state then heated to isotropic does indeed contract, as predicted. However, on cooling, the LCE will not form a polydomain but a monodomain with the same original orientation, and re-elongate back to its original shape. Thus, there must be some memory of the original direction encoded in the network that survives heating to isotropic and guides the director formed on cooling. Correspondingly, while such an LCE that is distorted in the nematic state will show anomalously soft elasticity, coupled to director rotation, it will not ideally soft. Rather, director rotation now requires a low but finite stress [28], as the network has a modest energetic preference for the director imprinted at crosslinking, ${\mathbf{N}}_{0}$.

Thus, even in the monodomain LCEs, to explain global actuation we must look beyond the very idealized microscopic model above. In practice, cross-linking in a nematic state leads to the director at crosslinking, ${\mathbf{N}}_{0}$ (naturally a reference state quantity) being imprinted permanently on the network, leading to several “non-ideal” effects including an energetic preference for ${\mathbf{N}}$ to align with ${\mathbf{N}}_{0}$ (breaking the degeneracy of the soft modes), a broadening the phase transition, and a residual para-nematic order above the transition temperature. Such non ideal effects are expected to be mild, given they are zero in a reasonable ideal model, but they are nevertheless crucial to describing actuation. Microscopically, such anchoring effects can be traced back to a variety of sources, including compositional fluctuations, finite length chains and rod-like crosslinkers, [43–45], and may be included in the elastic model by adding an anchoring term proportional to a small dimensionless non-ideality parameter $\alpha \ll 1$

7 $$ W_{NI}(\Lambda ) = \min _{{\mathbf{n}}} \left ( W_{NH}( \Lambda _{s}^{-1}({ \mathbf{n}}) \cdot \Lambda )+\alpha \mathrm{Tr}({\mathbf{n}}{ \mathbf{n}}\Lambda (I-{\mathbf{N}}_{0}{\mathbf{N}}_{0}) \Lambda ^{T}) \right ). $$

We note that above, we have reverted to the traditional “trace-formula” formulation in terms of ${\mathbf{n}}$ rather than ${\mathbf{N}}$ for consistency with prior literature. Critically, this energy has a unique global minimum with ${\mathbf{N}}={\mathbf{N}}_{0}$ and corresponding deformation $R\Lambda _{s}({\mathbf{N}}_{0})$, and final director ${\mathbf{n}}=R {\mathbf{N}}_{0}$ (for any rotation $R$), verifying an energetic preference for the director present at crosslinking. Conceptually, it can be thought of as slightly tipping the “Mexican-hat,” so that one unique position in the brim becomes the global minimum, although rolling around the brim (previously a zero energy mode) remains very low energy ($\mathcal{O}(\alpha )$) compared to other types of deformation.

The microscopic origin of mechanical shape-programming is now clear. The initial deformation during mechanical programming is accommodated at zero energy by a microstructure with an inhomogeneous director pattern. The corresponding local deformation from the isotropic state is mechanically compatible and everywhere in $K^{0}$. Second cross-linking increases the shear modulus, has little effect on $\lambda _{s}$ and hence $K^{qc}$, but does introduces a small non-ideal term creating a mild local preference for the director pattern in this particular microstructure, breaking the overall degeneracy. On heating back to the isotropic, the sample can simply revert to the original isotropic reference state, locally and globally. Critically, this works because the $\lambda _{s}$ that applied during the original formation of the microstructure is unchanged by second crosslinking, and hence is still reversed successfully on heating. Finally, on subsequent re-cooling, there is now an imprinted microstructural director pattern ${\mathbf{N}}_{0}$ that breaks the original degeneracy and guides the LCE back to a unique global minimum energy conformation with exactly the local and global deformations of the originally programmed state.

## Limits of Programming

The above understanding of the microstructural basis of mechanical programming makes a simple prediction. Programming deformations within $K^{qc}$ are accommodated by zero energy microstructure, leading to pseudo-plastic deformations before second cross-linking, and successful shape programming after second cross-linking. In contrast, programming deformations that are not in $K^{qc}$ are resisted elastically, even in the supersoft isotropic genesis polydomain state, and will not be successfully programmed upon second-crosslinking.

To probe this experimentally, we prepare the isotropic genesis polydomain LCE samples in thin sheets in the $x-y$ plane. We then attempt to programme various macroscopic states of 2D ($x-y$) stretch, with the stretch in the thickness ($z$) direction following from incompressibility, such that the programming deformation is:

8 $$\Lambda^{p}=\left(\begin{array}{ccc} \Lambda_{xx} & 0 & 0\\ 0 & \Lambda_{yy} & 0\\ 0 & 0 & \frac{1}{\Lambda_{xx}\Lambda_{yy}} \end{array}\right).$$

Given the samples are sheets, we can only apply tensile stresses in plane, limiting us to programming deformations in which the sheet becomes thinner rather than thicker, i.e. $\Lambda _{xx} \Lambda _{yy} \ge 1$. However, since the original sample is macroscopically isotropic, this set of deformations spans the entire set of physically distinct deformations in $\Lambda _{1}-\Lambda _{3}$ space.

The key experimental challenge is to impose these programming deformations in a uniform way. An initial attempt at imposing uniaxial stretches with $\Lambda _{xx}<\lambda _{s}$ by simply pulling on opposing ends of the strip quickly revealed that such samples tend to strain highly heterogeneously with some large regions taking up high strain and becoming clear, indicating full director reorientation, and others not straining at all (see photos in Fig. 1b.) To obtain uniform uniaxial strain fields, we thus first stretched the polydomain sample into a well aligned monodomain LCE with $\Lambda _{xx} = \lambda _{s} \approx 2$ everywhere. Next the LCE was placed on a hot plate with a temperature above room temperature but below the $T_{NI}$, which reduced the order parameter and caused a partial but uniform contraction of the LCE into a state with uniform strain of $\Lambda _{xx}<\lambda _{s}$. Such a sample still has monodomain orientation, and, if cooled, will re-elongate. We thus then trapped the hot LCE film between glass slides to prevent any further macroscopic strain, cooled the sample back to room temperature, and then UV-crosslinked.

The above strategy for imposing homogeneous macroscopic strains is limited to uniaxial deformations. To further achieve more general biaxial states, we adhered the polydomain LCE to a stretchable elastic substrate with significantly higher modulus/thickness than the LCE itself. We then defomed the LCE/substrate pair uniaxially or biaxially by simple pulling from the edges, which lead to an extended region of uniform macroscopic strain in the center of the LCE/substrate pair, owing to the substrates dominant elastic energy and quasi-convex energy function. We then UV-cured under strain, removed the LCE from the substrate, and cut-out the central region of uniform strain.

After second (UV) cross-linking, each sample was heated to the isotropic, and then cooled back to the nematic, to observe the resultant shape changes, and hence the quality of the programming. Precisely, we define $\Lambda ^{I}$ as the observed deformation from the original polydomain state to the new hot isotropic state (observed on heating), and $\Lambda ^{N}$ as the deformation from the original polydomain state to the new nematic state (observed on cooling). Perfect programming would give $\Lambda ^{I}=I$ and $\Lambda ^{N}=\Lambda ^{p}$. Given all our deformations are diagonal in the $x-y$ frame of the lab, our observations thus give us four directional measures of programming quality, which are the ratios of the observed and programmed deformations of the hot cold states ($\Lambda ^{I}_{xx}/1$, $\Lambda ^{I}_{yy}/1$, $\Lambda ^{N}_{xx}/\Lambda _{xx}$ and $\Lambda ^{N}_{yy}/\Lambda _{yy}$) all of which should be one for perfect programming. We combine these into a single figure of merit, fixity, by computing the four ratios, inverting any that are larger than one, and then taking the product. This figure of merit is necessarily somewhat heuristic, but has the essential property that it is one if and only if the sample perfectly actuates between the original hot and programmed cold shape, and always falls below one otherwise.

**Fig. 3:**
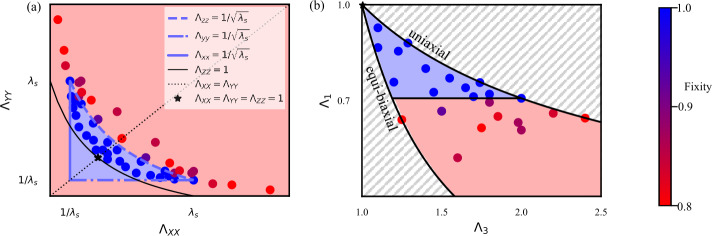
Limits of LCE programmability. (a) Mechanically programmed LCEs with various $\Lambda _{xx}$ and $\Lambda _{yy}$ values experimentally imposed prior to the second crosslinking. Color corresponds to fixity where 1 (blue) indicates successful programming and $fixity < 1$ (purple/red) indicates the sample either does not fully elongate to its programmed shape and/or does not completely contract to its original shape. Given the isotropy of the unprogrammed state, each experiment also appears mirrored in the $\Lambda _{xx}=\Lambda _{yy}$ line. (b) Re-plotting of the data in (a) as a function of the largest and smallest imposed stretches ($\Lambda _{1}$, $\Lambda _{3}$). In both plots, the shaded blue and red regions correspond to deformations inside and outside of $K^{qc}$, respectively, which is predicted to also be the region of programmability

The resultant data is shown in Fig. 3 (with additional images shown in Fig. S2), both in the experimentally intuitive $\Lambda _{xx}$, $\Lambda _{yy}$ space, and also replotted in $\Lambda _{1}-\Lambda _{3}$ space to match Fig. 2. In both cases, we see clearly that indeed programming deformations within $K^{qc}$ (blue region) have high fixity, while those beyond $K^{qc}$ (red region) do not, validating our central hypothesis.

## Microstructure Formation During Programming

**Fig. 4:**
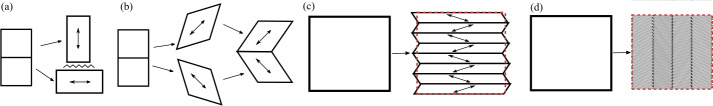
Stripe domain formation in LCEs. In each figure a hot isotropic state (left) transitions to a cold nematic state (right) with a microstructure. (a) An LCE in its isotropic state (left) cools to nematic (right), forming two domains with orthogonal director. The domains elongate along their director, leading to a mismatch in length of the boundary. Such a domain pattern is incompatible, and will not be formed. (b) If the directors form such that they are bisected by the boundary, the two domains agree on the length, and material continuity may be restored by body rotations. This leads to a compatible domain pattern, with piece-wise constant director regions divided by a bisecting line (plane). (c) Repeating this construction leads to a laminar or stripe microstructure, in which the local deformations are everywhere in $K^{0}$, but the macroscopic (average) deformation is not. (d) In 3D, hierarchical double laminates (stripes of stripes) are required to accomodate macroscopic deformations with all three principle stretches different to $K^{0}$, including the self accommodating microstructure that gives no macroscopic deformation

Our original discussion of the microstructures that form during mechanical programming avoided detailing exactly what form they take. In general, upon cooling from isotropic to nematic-polydomain, the microstructure formation takes place under a strong constraint of geometric compatibility. Not only must all the deformations be locally in $K^{0}$, but the resultant pattern of deformations must be the gradient of a displacement field $\Lambda =\nabla \mathbf{r}$. This condition has no counterpart in the liquid state, and is not obeyed by the schlieren textures characteristic of liquid nematic polydomains. A schematic of the compatibility problem is given in Fig. 4(a), where two neighboring domains form with orthogonal directors, leading to disagreement about the length of the boundary between the two domains. However, a different situation arrises if the boundary between the domains bisects the two directors. In this case (Fig. 4(b)), the two domains agree on the length of the interface, and material continuity can be restored by a simple body rotations. Periodically repeating this construction leads to stripe-domain microstructures (laminates) that oscillate between piece-wise constant directors/deformations that differ by a shear (Rank-1 comparability, (Fig. 4(c))). Such stripe-domains/laminates have been observed in monodomain LCEs [28], and are common in shape-memory alloys [38].

The theoretical relaxation of $W_{N}(\Lambda ) \to W^{qc}(\Lambda )$ presented in [29] provides an explicit construction for $W^{qc}(\Lambda )$ using double-laminates — i.e. a second generation of stripes/lamination between regions that individually contain first-generation stripes/laminations (Fig. 4(d)). Such double laminations are invoked throughout $K^{qc}$, with the exception of $K^{0}$ (monodomain) and the horizontal boundary of $K^{qc}$ at $\Lambda _{1}=1/\sqrt{\lambda _{s}}$ (single laminate). However, despite this ubiquity throughout $K^{qc}$, hierarchical stripes have not been observed in LCEs, and the mathematical treatment provides no guarantee that they are required to relax down to $W^{qc}(\Lambda )$, only that they are sufficient. Furthermore, given the ideal LCE energy $W_{N}(\Lambda )$ contains a continuously connected set of low energy states (as opposed to the discrete, separated energy wells associated with martensite variants in shape memory alloys), it is far from clear that such piece-wise constant patterns are the only option: one can also imagine continuously varying director patterns, rather than piecewise constant. Regrettably, the actual experimental domain structures in isotropic genesis polydomains are typically too fine to be directly observed optically, though insights can be obtained via x-rays and light scattering [46].

It is thus interesting to look experimentally at what microstructures form within the LCEs during mechanical programming. In many cases of complex programming experiments (e.g. the seashell) all we are able to observe is an overall turbid polydmain appearance, suggesting a highly-scattering microstructure with micron-scale domains (c.f. $\sim 170\mu \mathrm{m}$ thickness) that defy resolution by optical microscopy. However, during our careful homogeneous-strain experiments with programming stretches in $K^{qc}$ (and contingent on using the optimized relative cross-link densities of the first and second network reported by *Barnes and Verduzco* [14]) we are nevertheless typically to directly observe regions of complex cross-hatch type microstructure within the LCEs under cross-polarized transmission optical microscopy.

To obtain clear images, we require microstructures that are both relatively homogeneous and coarse. In our case, the clearest images were obtained in uniaxially programmed sample with $\Lambda _{xx}<1.7$, which is close to but less than the monodomain strain of $\Lambda _{xx}\approx 2$. Fabrication followed the previously described protocol for homogeneous uni-axial programming, except, to promote a homogeneous microstructure, a additional step was added in which the sample was heated to isotropic and then cooled to room temperature whilst between the glass slides but before UV cross-linking, so that the microstructural pattern was selected on cooling from isotropic; this process mirrors the acceleration of director rotation discussed in Fig. 1(c). The choice of $\Lambda _{xx}<1.7$ was found to be advantageous for two reasons. Firstly, it produces a pattern of domains in which all directors lie fairly close to the $x$ axis, reducing the optical contrast between domains and hence the overall level of scattering. Secondly, the large programming strain provide a global frame of reference to orient the microstructure consistently throughout the sample (including in the thickness direction), which contrasts with $\Lambda _{xx}=1$, for which all orientations of a given microstructure would be equivalent.

**Fig. 5:**
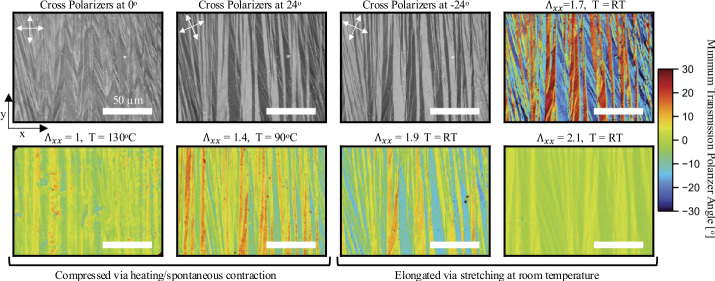
Optical images of an LCE film ($t = 170 \mu m$ with a programmed $\Lambda _{xx} = 1.7$ along $n_{o}$ resulting in a double laminate microstructure. Unstretched, at room temperature the cross-polarizers were rotated from -45 to +45^*o*^ relative to $n_{o}$, to obtain an image with a colormap denoting the polarizer angle that resulted in the minimum transmission value for each pixel. Similar images are also included when the LCE is heated, resulting in spontaneous contraction, as well as mechanical stretching at room temperature

Transmission POM images of the resulting microstructure are shown in Fig. 5, which confirms the existence of double laminates as a physically observed LCE microstructure. In particular, when the sample is viewed under cross-polarizers oriented parallel/perpendicularly to the programmed deformation ($0^{\circ}$), one can see the small-scale second-generation of laminates which assemble, herringbone-like, into the larger first-generation. Conversely, rotating the polarizers $\pm 24 ^{\circ}$ removes the contrast between domains in the second-generation, and highlights the contrasts between large first-generation laminates, leading to images that are reminiscent of classic single-generation stripe domains. The large-scale lamination’s have a characteristic widths $\approx 10\mu m$ while second generation stripes have smaller widths $\approx 2 \mu m$, which is indeed approaching the limit of resolvability in the microscope. Furthermore, both widths are considerably smaller than the sample thickness ($170 \mu m$), highlighting that the microstructure must indeed be very uniform in the thickness direction to be observed in transmission POM.

To gather quantitative information on the director profile of the microstructure we systematically rotated the cross-polarizers by $2^{o}$ increments, overlaid the images, determined which angle minimizes the light transmission at each pixel, and used this data to reconstructed the image with the color corresponding to the angle of the polarizers relative to the $x$ axis. This data thus provides information about the (in-plane, thickness averaged) director in each pixel. More precisely, at angles close to $0^{\circ}$, this local director is aligned either along or perpendicular to the $x$ axis, and similar for other angles. The resultant false-colour image clearly highlight the two generations of laminations.

We further observed the microstructure evolution in the sample during heating (with corresponding spontaneous global contraction, images at $\Lambda _{xx} =1.4$, $\Lambda _{xx} =1$), and also during additional mechanical stretching at room temperature (images at $\Lambda _{xx} =1.9$, $\Lambda _{xx} =2.1$). As might be expected, during room temperature stretching, Fig. 5 shows the basic domain pattern remains unchanged, but all the individual directors rotate towards $0^{o}$, which is the final monodomain state. More surprisingly, similar director rotation towards the $x$ direction is also observed on heating, suggesting that during heating not only is the order parameter decreasing, but there is also a degree of reorientation. This apparant rotation is likely due to the advection of the director with the deformation as the microstructural shears/rotations are undone (see Fig. 4b), rather than true director rotation with respect to the LCE matrix. Indeed, an elementary 2D calculation for a single laminar (stripe) structure with interfaces along $y$ and imprinted director making angles $\pm \theta $ with $x$ in the isotropic state suggest that on cooling, the director will be advected to the larger angle $\theta _{N}=\tan ^{-1}(\lambda _{s}^{3/2} \tan{\theta}))$, and we are likely observing the reverse of this process on heating.

**Fig. 6:**
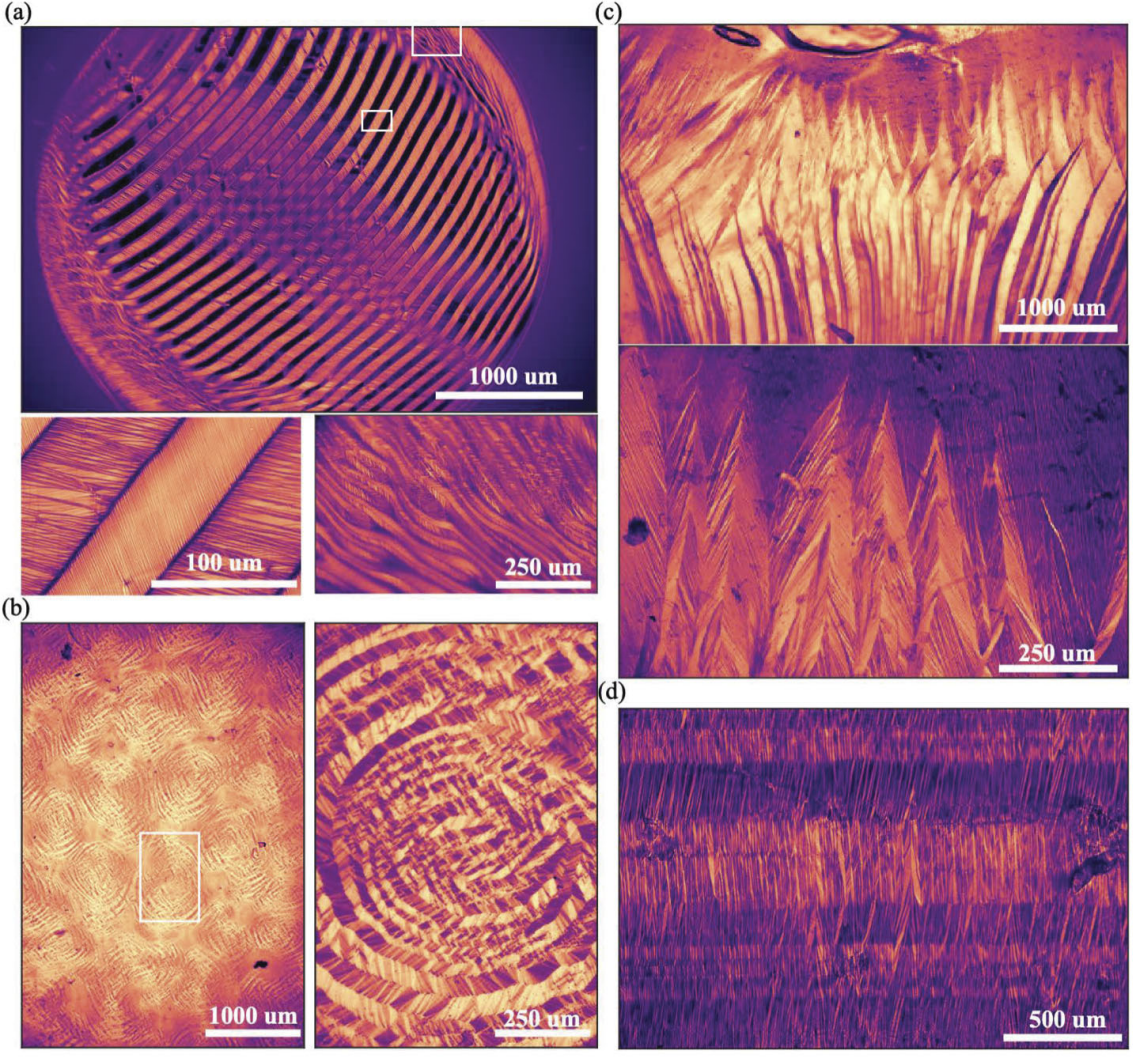
Gallery of experimentally observed microstructures in LCEs under-cross-polarizers. (a) An LCE blister exhibiting large regions of uniform double-lamination as well as double-laminates with curved boundaries towards the edges of the blister. (b) A sequentially bi-axially stretched LCE in semi-soft clamps resulting in sharp peaks of double laminates formed close to the edge of the sample. (c) An LCE film that was deswollen while adhered on one side to a glass slide exhibiting regular circular features of double-laminates. (d) A sequentially bi-axially stretched LCE imaged in the middle of the sample with triple-laminates

In the course of our investigations, we have observed many other occurrences of visually striking microstructures within LCEs, and we present the highlights as a showcase in Fig. 6. At this stage, the mechanics that select the type and scale of microstructure in these experiments remains unclear, but the gallery clearly shows several additional examples of hierarchical lamination, along with some other new qualitative features that we can rationalize.

First, in Fig. 6a, we see an LCE blister ($t = 85\mu m$) that rises proud in a film that is otherwise adhered to a glass substrate. To form the blister, the initial network was formed between two glass slides. The top slide was then removed, and the lower slide and LCE were placed on a hot plate ($T=150^{o}$C) to rapidly and violently evaporate the chloroform solvent, with the blisters appearing due to the formation of pockets of gas. (In contrast, the normal protocol would be to remove both glass slides and evaporate the solvent slowly, resulting in uniform contraction of the network into the isotropic genesis polydomain state.) When heated and cooled, large stripes appear along the blisters with a second fine generation of lamination also clearly visible within. The lamination boundaries also curve towards the edge of the blister. The much larger scale of these microstructures likely originates from their comparatively loose clamping: the blisters are only clamped at the perimeter, whereas our previous observations were clamped in the thickness direction during programming. Furthermore, repeated experiments with directional cooling (Fig. S3, S4, M1) reveal that the first lamination stripes always arise parallel to the cooling front, suggesting a dynamic contribution to pattern selection. Conversely, if a similar sample is deswolen slowly at room temperature while adhered to the glass slide, no blisters form, but, on one (non reproducible) occasion we observed a pattern circular double laminates in the adhered film (Fig. 6b). These circular interfaces perhaps point to the increased possibilities of the continuously reorientable director, vs the discrete energy wells of martensite/austenite systems.

In Fig. 6c a free-standing polydomain LCE film was sequentially stretched in orthogonal directions. As expected, coarse double lamination occurs in the bulk of the material, reflecting the deformation lying in $K^{qc}$, and the thickness direction not being clamped. In this case, the sequential stretching perhaps provides the global orientation for the microstructure. Additionally, towards the edges of the sample where the laminated regions reach the clamps, we observe a much finer microstructures that recursively branch and coarsen into the bulk. Similar structures have been seen in shape memory alloys [47], where branching sequences are known to enable a better fit to the clamped boundary while reducing interfacial penalties in the bulk. Finally, in Fig. 6d an LCE film was sequentially orthogonally strained three times ($0^{o}$, $90^{o}$, $0^{o}$C) resulting a plaid-like microstructure in the center of the film exhibiting third-order lamination.

We hope these preliminary results on the different types of microstructe observed during programming motivate additional research towards a predictive framework!

## Soft Modes of Programmed LCEs

Finally, we briefly investigate the mechanical properties of mechanically programmed LCEs. In general, we expect mechanically programmed LCEs to still exhibit director rotation and (non-ideal) soft elasticity in response to imposed strains, just as monodomain LCEs do. More precisely, the set of deformations in $K^{qc}$ are still all expected to be accessible at very low energy (via director rotation and rearrangement of the microstructure) while any deformation that takes the LCE out of $K^{qc}$ will be resisted by a substantial elastic stress. In this sense, all mechanically programmed LCEs have access to exactly the same set of soft modes, described by $K^{qc}$. However, importantly, $K^{qc}$ is described in terms of deformations $\Lambda $ from the original isotropic state, while the effect of mechanical programming is to “fix” a particular different member of $K^{qc}$, $\Lambda ^{p}$, as the preferred shape in the nematic state, and the set of all soft deformations may appear very different with respect to this programmed state, even though it is always the same with respect to the isotropic state. For example, a monodomain is soft when pulled transversely, but hard when pulled longitudinally, while a $\Lambda ^{p}=I$ polydomain is soft in every direction.

More precisely, if a sample is prepared with programming deformation $\Lambda ^{p} \in K^{qc}$, and then subject to a further deformation gradient $\lambda $ with respect to this relaxed nematic shape, the total deformation from the isotropic is $\Lambda =\lambda \cdot \Lambda ^{p}$, and the deformation $\lambda $ will be soft if $\lambda \cdot \Lambda ^{p} \in K^{qc}$. The set of deformations $\lambda $ that can be achieved softly thus clearly depends on $\Lambda ^{p}$. This provides an opportunity to use mechanical programming to engineer the anisotropy of this soft set.

As a simple illustration of this idea, we again create a set of samples in thin sheet like geometries in the $x-y$ plane, programmed with macroscopic states of 2D ($x-y$) stretch, with the stretch in the thickness ($z$) direction following from incompressibility:

9 $$\Lambda^{p}=\left(\begin{array}{ccc} \Lambda_{xx} & 0 & 0\\ 0 & \Lambda_{yy} & 0\\ 0 & 0 & \frac{1}{\Lambda_{xx}\Lambda_{yy}} \end{array}\right).$$

**Fig. 7:**
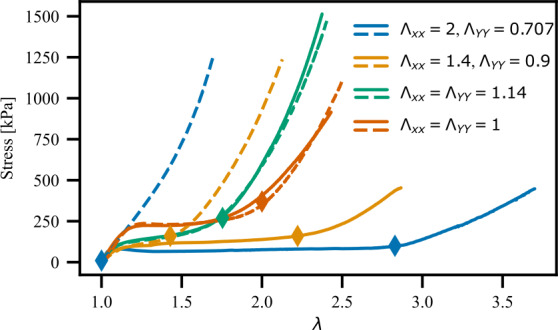
Stress-Extension curves for LCEs with varying $\Lambda _{xx}$ and $\Lambda _{yy}$. Each programming strain was tested under uniaxial extension in the $\lambda _{xx}$ direction (dashed line) and the $\lambda _{yy}$ direction (solid line) at a strain rate of 0.25%/min. Diamonds indicate the theoretical end of the soft-behavior

We limit our attention to $\Lambda ^{p} \in K^{qc}$, so that all are successful examples of shape programming. We then test these samples mechanical response to uniaxial elongation in the $x$ and $y$ directions (separately), to get the set of stress-strain curves shown in Fig. 7. (More precisely, we cut separate rectangles that are elongated in the $x$ and $y$ direction respectively, and then stretch them longitudinally using an Instron tensile machine.)

From the resultant data, we confirm that all samples show a (non-ideal) soft response, with a substantial low-stress plateau associated with director rotation. However, the extent of this plateau, and its division between the $x$ and $y$ stretching curves varies considerably between samples. As expected, the monodomain programmed sample has the longest plateau for stretching transverse to the director, and none for longitudinal, while other samples split the soft behavior to differing degrees between the two directions.

To compare the results with our theory, we consider that, during testing, the total deformation from the isotropic state is of the form

10 $$\Lambda =\left(\begin{array}{ccc} \lambda_{xx} & 0 & 0\\ 0 & \lambda_{yy} & 0\\ 0 & 0 & \frac{1}{\lambda_{xx}\lambda_{zz}} \end{array}\right)\cdot \left(\begin{array}{ccc} \Lambda_{xx} & 0 & 0\\ 0 & \Lambda_{yy} & 0\\ 0 & 0 & \frac{1}{\Lambda_{xx}\Lambda_{zz}} \end{array}\right).$$

This total deformation will exit $K^{qc}$ when it describes a monodomain aligned in the stretching direction imposed during the test. For a test stretching in $x$, this requires:

$$ \Lambda _{3} = \lambda _{xx} \Lambda _{xx} = \lambda _{s} \implies \lambda _{xx} =\frac{\lambda _{s}}{\Lambda _{xx}}. $$

At this point, on exiting the soft deformations, the corresponding $y$ deformation is expected to be $\lambda{_{yy}}=1/(\Lambda _{yy} \sqrt{\lambda _{s}})$. Similarly, for testing with elongation in the $y$ direction, soft behavior will finish when $\lambda _{yy}=\lambda _{s}/\Lambda _{yy}$. These predicted strain points for the end of soft behavior are marked on each of the stress-strain curves with a star, with the locations agreeing well with the apparent end of the stress plateau.

## Discussion

In this paper, we have clarified the link between the “supersoft” elasticity of isotropic genesis polydomain LCEs and the direct mechanical shape programming of LCEs that allows arbitrary complex morphing on actuation. The key idea is that the isotropic-nematic phase transition endows an isotropic genesis polydomain with a true “Mexican-hat” style elastic energy, with a large brim of global minimizers with different deformations and director orientations. Furthermore, through the use of microstructural deformations, all macroscopic deformations within this brim (formally, in $K^{qc}$, which includes identity) can also be achieved with zero energy. The mechanical response of such polydomains within this large region of deformation space can thus be described as liquid like: there is no restoring elastic stress at all, and samples deformed within $K^{qc}$ do not tend to recover their shape. However, on heating back to isotropic, the energy reverts to a standard neo-Hookean with the minima at identity, and the sample recovers its shape. During mechanical programming, the role of second cross-linking is simply to break the degeneracy of $K^{qc}$, imprinting one microstructure and therefore one deformation as the preferred one on cooling. In contrast, if one attempted mechanical programming with a nematic genesis polydomain as the intermediate state (with otherwise identical composition and history), the macroscopic energy for the polydomain will now have a single global minimum at identity ($K^{qc}$ shrinks to a point) [35, 36], and, although mechanical programming may still be partially successful, faithful mechanical programming in which the programmed deformation is exactly reversed on heating will not be possible.

LCEs that are mechanically programmed with complex deformations during actuation are thus directly analogous to their actuating monodomain counterparts, but with a complex microstrucural director profile imprinted, rather than a monodomain one. Accordingly, in their nematic state, they also exhibit soft elasticity, with the soft set of deformations mapping back to the $K^{qc}$ of the original isotropic genesis polydomain. Moreover, as in the monodomain case, the imprinting process breaks the perfect degeneracy of $K^{qc}$, introducing a degree of non-ideality into this mechanical softness that manifests as semi-soft stress plateaus during director rotation.

The success of mechanical programming lies in the fact that $K^{qc}$ contains all incompressible deformations that are in the vicinity of $\Lambda =I$. In other words, $K^{qc}$ includes all modest deformations. Therefore, any complex shape morphing can be achieved, provided there is a map from the reference shape to the target shape that is everywhere in $K^{qc}$, which is guaranteed to be true for any sufficiently mild shape-programming problem. Furthermore, any incompressible deformation can be brought within $K^{qc}$ by making $\lambda _{s}$ large enough. Hence, given a target shape, a reference state, and a candidate incompressible map between them, provided only that the deformations in the map are not singular, it will always be possible to choose a $\lambda _{s}$ large enough that all the deformations are in $K^{qc}$, and hence the desired shape change is programmable — though, obviously, in practice $\lambda _{s}$ is a material property, placing limits on what a given material can do.

The observations about shape-programming working for all mild deformations also provide clarity about what shape changes can and cannot be programmed analytically. Here the result is very heartening. LCEs, with their one degree of freedom (director angle) but varied in full 3D, are capable of producing essentially arbitrary complex shape changes. There is perhaps an analogy to the Nash embedding theorem in differential geometry. Just as Nash guarantees that, if you can embed a target domain into Euclidian space with a map that only shortens distances, you can embed it isometrically using, essentially, hierarchical wrinkles, here we can guarantee that if you can map a target domain to a reference domain with a (isochoric) map that everywhere has principle stretches less extreme that $K^{0}$, you can also find a hierarchical (microstructural) map that is everywhere exactly $K^{0}$.

We now understand that mechanical shape programming indeed works simply by sculpting a preferred director profile in the material, much as the more analytical approaches to complex shape programming work by explicitly designing a director profile and then fabricating it. The success of mechanical shape programming may thus offer insights into director design. The connections are necessarily a little distant, as mechanical programming allows 3D director structures and orientations, while most fabrication techniques (and hence analytic director designs) focus on in-plane directors. However, despite this, one can directly imagine tackling the inverse problem numerically by explicitly mimicking mechanical programming. For example, to solve the “metric programming” problem of morphing a flat sheet into a given curved shell, one could start by defining the curved shell in finite elements, endowing it with an elastic energy $W_{N}$ (exhibiting the soft mode) flattening it *in silico* into the plane, and allowing energy minimization to find the lowest energy state. This computation is exactly what would be done to simulate a polydomain LCE being flattened into the plane. If minimization produces a zero energy state, the deformation is everywhere in $K^{0}$, and one can then read off the corresponding director pattern that will morph the flat domain into the curved surface on heating. In hindsight, the algorithm deployed in [11] works in a very similar way to this (although without making the link to a physical energy/problem). Alternatively, one could take a similar approach with $W^{qc}$ rather than $W^{N}$, and then, after finding a zero-energy state, reconstructing the full director profile microstrucurally using the known microstructures that create each element in $K^{qc}$. Indeed, such hierarchical designs have already been deployed in pneumatic systems in [48], although not via such a design process.

In both cases, the constraint that the director lie in plane, and only vary in plane, can be implemented by modifying the energy that is used. The $W_{N}$ discussed here is the full 3D form describing real LCEs, but it may easily be replaced by an artificial 2D form (that constricts the director to the plane), and/or its microstructural relaxation. Similarly, beyond LCEs, there are many other shape programming systems that do not literally have soft modes. For example, a flat gel sheet with a crosslink density that is patterned in the plane will dialate with a patterned swelling factor on solvation, leading to a palate of spontaneous distortions $\Lambda _{s}(\alpha ) = \alpha I$, where $\alpha (x,y)$ is designed via crosslink density, and implements a conformal map on swelling [7, 49, 50]. In this system, there is no literal soft mode: in a given solvent the swelling of the gel is fixed, and will deform isochorically to subsequent deformations. However, one may still imagine a material with energy $\min _{\alpha}W_{NH}(\Lambda \Lambda _{s}^{-1}(\alpha ))$ that can traverse the set of spontaneous deformations at zero energy, and an FEM implementation of such a material could be used to tackle pattern design in the same way. Indeed, this numerical approach was very recently demonstrated to tackle complex morphing of LCEs via patterned stimulation rather than patterned director [13].

Returning to LCEs, we see several avenues for further work. Firstly, we have here focused attention on programming deformations within $K^{qc}$, as those are the ones which are faithfully reversed on heating. However, it will be interesting to examine the results of fixing deformations beyond $K^{qc}$. In such cases the isotropic genesis polydomain bears elastic stresses during the second cross-linking, and the polymers are extended beyond the degree prefered by the nematic phase. Such samples are thus different at a network level to the ones considered here, and likely have correspondingly different transition temperatures, and degrees of non-ideality. There is also a pressing need for a satisfactory statistical picture of such double-network LCEs, which may cast light on how to balance the cross-link density between the two rounds of crosslinking, and also how to predict the degree of anisotropic response.

It is also extremely interesting to look more closely at the actual microstructures observed in these materials. Here we have reported preliminary results showing the occurrence of double-laminate structures, and a gallery of fascinating and beautiful patterns encountered in different programming scenarios. However, despite these observations, we still lack a framework for predicting what patterns are selected in a given scenario, and at what length scale they emerge. Candidate factors that influence microstructure selection include sample size, confinement type, cooling rate, spatial cooling gradient and viscous effects, and dissecting these factors is likely to be a substantial and rewarding endeavour.

Finally, we observe that the soft elasticity and director rotation of LCEs has long been an object of curiosity, but application focused efforts have largely targeted actuation. Here, we see that soft elasticity enables facile mechanical programming, practically removing almost all limits on what complex morphing is possible (within volume conservation). Soft elasticity thus enables LCEs to solve the problem of designing a director profile for a given transformation, and with a degree of sophistication and success that defies current analytic + fabrication methods. In this sense, soft elasticity can also be thought of as making the LCE a form of mechanical computer, or even endowing it with a degree of intelligence. It seems likely that physical materials with different soft modes may similarly be used to solve other problems, perhaps providing an interesting general framework for mechanical computation.

## Supplementary Information

Below are the links to the electronic supplementary material.

(PDF 5.0 MB)

(MP4 13.5 MB)

## Data Availability

No datasets were generated or analysed during the current study.
