# Supplementary material for: Microstructural Basis of Complex Mechanical Programming in Liquid Crystal Elastomers
Source: J Elast. 2025 May 29;157(3):48. doi: 10.1007/s10659-025-10138-4 (PMC12122613; doi:10.1007/s10659-025-10138-4)
Supplement: Supplementary file 1 — (PDF 5.0 MB) [file 10659_2025_10138_MOESM1_ESM.pdf]

Supplemental Material: Microstructural basis of complex mechanical programming in liquid crystal elastomers

Morgan Barnes and John S. Biggins

May 15, 2025

1 I. Experimental Details

1.1 Materials

2-Methyl-1,4-phenylene bis(4-(3-(acryloyloxy)propoxy)benzoate (RM257) was purchased from Daken Chemicals, 2,2'-(Ethylenedioxy)diethanethiol(EDDET), pentaerythritol tetrakis(3-mercaptopropionate) (PETMP), dipropylamine (DPA), (2-hydroxyethoxy)-2- methylpropiophenone (HHMP), and chloroform were purchased from Merck. Stretchable double sided mounting tape was purchased from Amazon. All materials were used as received.

1.2 LCE Synthesis

LCEs were synthesized following the same procedure reported previously. [1] Briefly, 1 mmol of RM257 (588 mg), 0.68 mmol of EDDET (12.09 mg), 0.11 mmol of PETMP (55.45 mg) and 0.588 ml of chloroform were added into a glass vial, gently heated with a heat gun, and vortexed until all components were dissolved and mixed. This composition has 10% excess acrylates compared to thiols and 25% of the thiols come from PETMP. Next, 180 mg of a 2 wt% solution of DPA in chloroform was added to the vial to catalyze the first crosslinking and further mixed. The LCE was then deposited onto a glass slide with plastic spacers to control the film height, and a second glass slide was placed on top of the solution. After 3 hours of curing the LCE was removed from the glass slides and heated on a 100°C hotplate for 30 minutes or until the sample was completely opaque when cooled, indicating removal of the solvent resulting in a polydomain LCE. The LCEs were then mechanically deformed in a variety of ways detailed below and UV cured with 365 nm light for 10 minutes to form the second network.

1.3 Programming Shape Changes

The LCE seashell was made by lightly pressing the LCE on top of a seashell, and wrapping it around the edges prior to UV curing. As the LCE deforms more readily and contracts when heated close the T_{NI}, the LCE was partially heated with the heat gun while wrapped onto the LCE to either better mimic the topographical features of the LCEs or to contract areas that were over stretched during handling.

To program uniaxial stretches that were less that λ_s with a uniform strain throughout, samples were stretched to λ_s and then placed on a a glass slide, and then put on a hot plate heated to temperatures greater than room temperature but less then T_{NI} resulting in uniform, partial contraction of the LCE. A glass slide was then placed on top of the LCE to act as a light clamp and then the glass slides/sample was taken off the hot plate and cooled to room temperature to achieve microstructures and uniform uniaxial strains less than λ_s. Microstructure appeaed upon cooling from $T < T_{NI}$ temperatures as well as when cooled from the isotropic, although slightly more uniform microstructure was present when cooling from the isotropic.

Biaxial stretching to determine the bounds of programmability was achieved by drawing a 1 cm x 1cm box on an LCE. The LCE was then placed on double sided stretchable mounting tape. The tape was then biaxial stretched to achieve uniform stretching of the LCE sample and the strain was calculated from the resulting size of the drawn box on the LCE.

1.4 Fixity Calculation

To calculate the overall fixity of mechanical programming we first the individual isotropic (contraction) and nematic (elongation) fixities, f^I and f^N in both the x and y directions as the ratio of the contracted/elongated lengths vs the original/programmed lengths.

$$f^I_{xx} = \frac{\Lambda^I_{xx}}{1} = \Lambda^I_{xx} \tag{S1}$$

$$f^I_{yy} = \frac{\Lambda^I_{yy}}{1} = \Lambda^I_{yy} \tag{S2}$$

$$f^N_{xx} = \frac{\Lambda^N_{xx}}{\Lambda)xx} \tag{S3}$$

$$f^N_{yy} = \frac{\Lambda^N_{yy}}{\Lambda_{yy}} \tag{S4}$$

The overall fixity is then defined as

$$f = {f^I_{xx}}^a * {f^I_{yy}}^b * {f^N_{xx}}^c * {f^N_{yy}}^d \tag{S5}$$

where $a, b, c, d = 1$ if $f^I_{xx}, f^I_{yy}, f^N_{xx} or f^N_{yy} < 1$ and $a, b, c, d = -1$ if $f^I_{xx}, f^I_{yy}, f^N_{xx} or f^N_{yy} > 1$. This is to ensure that $f = 1$ is only achieved for an ideally programmed LCE which fully contracts and elongates to its original/programmed shape and reduces if it fails to achieve that along any direction.

1.5 Tensile Testing

LCEs were cut into rectangular strips (0.6cm x 2 cm X 0.025 cm) and mounted into linear tension clamps on an Instron 68SC-05 with a 5 N load cell. Samples were stretched at a strain rate of 0.025, 0.25, 2.5, and 250%/min.

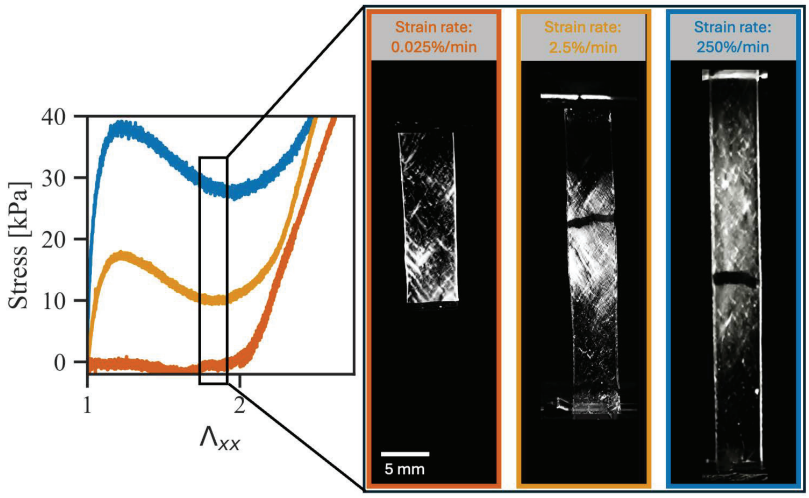

Figure S1: Images of LCEs prior to the second crosslinking during a tensile test in an Instron machine at different strain speeds. The sample images were all taken at $\Lambda_{xx} \approx 1.9$. The cross-hatch microstructure is coarser at slower strain rates.

1.6 Polarized Optical Microscopy

Cross-polarized optical micrographs were taken using a Nikon Eclipse LV100nd microscope in transmission mode with fixed cross-polarizer angles and a rotating sample stage. A a Linkam LTS420 hot stage was used for images taken a temperatures above room temperature.

To construct the image of minimum transmission angle of a sample, as shown in Figure 5 of the main text, the sample was equilibrated at a given temperature or strain and the orientation and the position that best showed the microstructure was imaged. Next, the sample was rotated by $\pm 2^o$ and imaged again until the final angle was $\pm 45^o$.

The stack of images was then processed in python using OpenCV. First, all images were digitially rotated so that they had the same orientation as the 0° image in a matrix i,j and converted to grayscale. The images were then stacked on top of each other to a form a single matrix of size i,j,m. A new matrix (i,j) was then constructed where the value was the orientation angle associated with the minimum transmission value at each pixel (i,j) in the (i,j,m) stack. The constructed matrix is then turned into a new image where colormap corresponds to the film angle associated with the minimum transmission value.

References

[1] Morgan Barnes and Rafael Verduzco. Direct shape programming of liquid crystal elastomers. *Soft Matter*, 15(5):870–879, 2019.

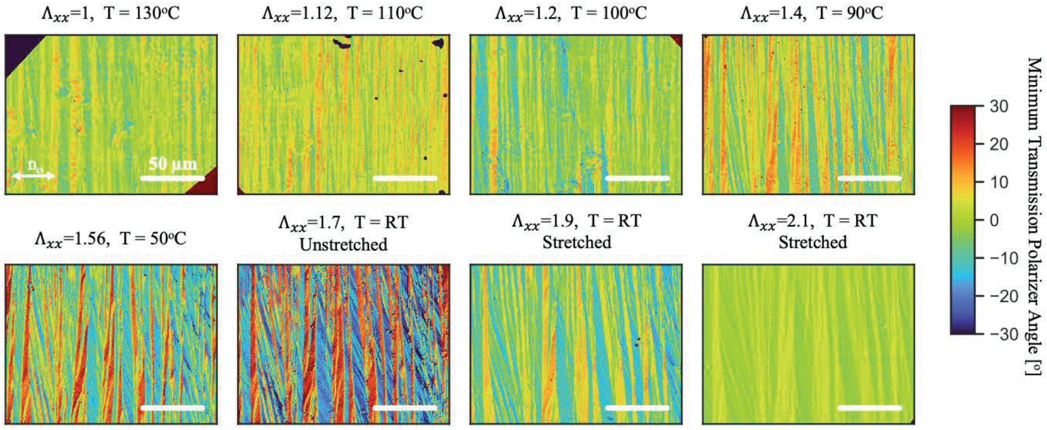

Figure S2: Data shown in Figure 5 of the main text with additional contracted lambda values as the film is heated.

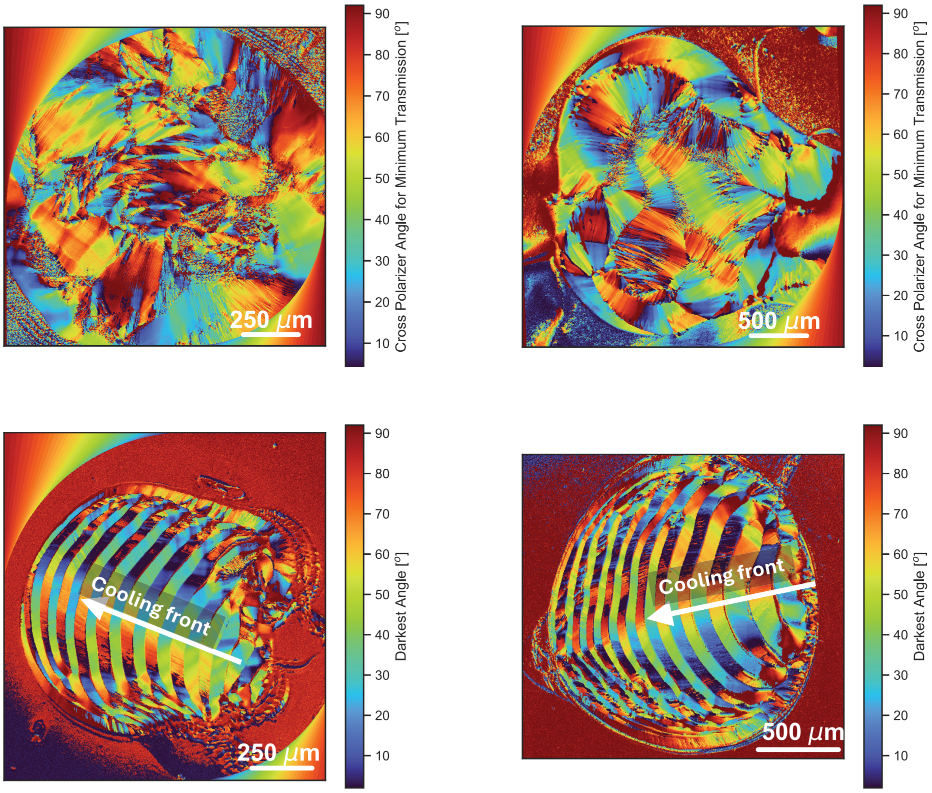

Figure S3: Image of LCE blisters with the colormap detailing the cross-polarizer angle that has minimum transmission value at each pixel. The top row showcases two blisters ($t \approx 50\mu m$) that were rapidly cooled from the outer edges in, resulting in defined director order but without any regular patterns. The bottom two images show similar blisters that were cooled directionally by sliding the hot sample/glass slide across a metal heat sink which results in regular stripes of alternating alignment. The spacing of the stripes can be controlled by changing the speed of the cooling front. Slower cooling fronts yielded large spacing as shown initially in the bottom right image. However, when the slide is swiped across the heat sink with increasing speed the stripes get closer together as they appear.

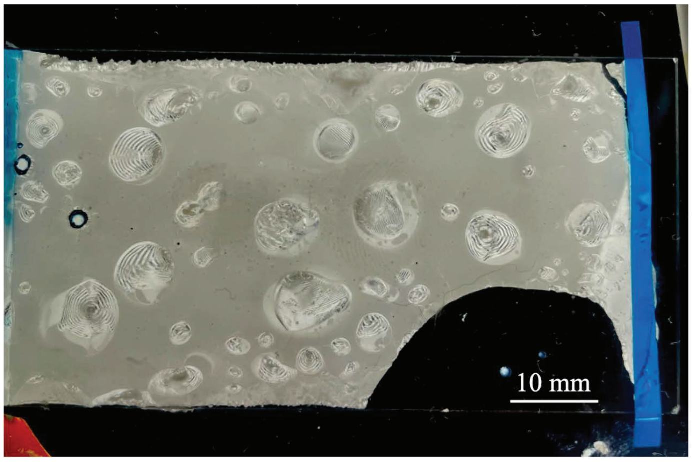

Figure S4: Photograph of multiple LCE blisters ($t = 100\mu m$) with microstructure on a glass slide.
